# Preliminary Interpretations of Epigenetic Profiling of Cord Blood in Preeclampsia

**DOI:** 10.3390/genes13050888

**Published:** 2022-05-16

**Authors:** Junrui Ma, Zhongqun Zhan, Ning Li, Yanli Huang, Yan Li, Lu Liu, Qi Shen, Qiao Chu, Xiaonan Wang, Benqing Wu, Hui Zhang

**Affiliations:** 1 School of Public Health, Shanghai Jiao Tong University School of Medicine, Shanghai 200025, China; 53586mjr@sjtu.edu.cn (J.M.); qiaochu@shsmu.edu.cn (Q.C.); xiaonanwang@shsmu.edu.cn (X.W.); 2 Faculty of Medical Laboratory Science, College of Health Science and Technology, Shanghai Jiao Tong University School of Medicine, Shanghai 200025, China; 3 Institute of Translational Medicine, University of Chinese Academy of Sciences Shenzhen Hospital, Shenzhen 518106, China; zzqun21@126.com (Z.Z.); 15813350609@139.com (Q.S.); 4 Cytotherapy Laboratory, The First Affiliated Hospital (Shenzhen People’s Hospital) Southern University of Science and Technology, Shenzhen 518020, China; lining.yatu@hotmail.com; 5 Department of Obstetrics, University of Chinese Academy of Sciences Shenzhen Hospital, Shenzhen 518106, China; 18126081431@sohu.com (Y.H.); zhangshi2001hs@163.com (Y.L.); ll18122069196@163.com (L.L.); 6 Department of Neonatology, Shenzhen Guangming Maternity & Child Healthcare Hospital, Shenzhen 518107, China

**Keywords:** preeclampsia, epigenetic alternation, immunomodulation, hypoxia response, *GATA* family transcription factor

## Abstract

Preeclampsia (PE) is characterized by new-onset hypertension after 20 weeks of pregnancy and results in high maternal and fetal mortality worldwide. It has been reported that PE is associated with abnormalities in the umbilical cord and cord blood. However, previous studies were focused primarily on the transcriptomics level, while the underlying gene regulatory landscapes are still unclear. Thus, we performed the Assay for Transposase-Accessible Chromatin with high-throughput sequencing (ATAC-seq) using the umbilical cord blood samples collected from a patient with superimposed PE and three healthy donors to uncover the chromatin accessibility changes attributed to PE. We have identified genes associated with immunomodulation and hypoxia response that have higher chromatin accessibility close to their transcription start sites. Motif analysis indicated that the *GATA* family transcription factor binding was enriched in PE and may play an essential regulatory role in the disease progression. Overall, our findings provide an overview of gene regulatory programs and the corresponding downstream pathways associated with PE that may influence the placenta function and fetal growth.

## 1. Introduction

Preeclampsia (PE), one of the leading causes of maternal and fetal mortality, is a pregnancy-associated complication characterized by new-onset hypertension and is often associated with proteinuria [1]. The International Society for the Study of Hypertension in Pregnancy (ISSHP) defines PE as de-novo hypertension (systolic blood pressure (SBP) ≥ 140 mmHg or diastolic blood pressure (DBP) ≥ 90 mmHg) present after 20 weeks of gestation accompanied by ≥1 of the following new-onset conditions: proteinuria (≥1+, 30 mg/dL; urine Protein/Creatinine Ratio (PCR) ≥ 30 mg/mmol (0.3 mg/mg)), material organ dysfunction, including acute kidney injury (creatinine ≥ 90 umol/L; 1 mg/dL), liver involvement, neurological complications, hematological complications, uteroplacental dysfunction [2,3]. Proteinuria is no longer required for a diagnosis of PE. The most life-threatening complication is HELLP (hemolysis, elevated liver enzymes, and low platelets) syndrome, leading to high mortality in patients with PE [4]. Depending on the gestational age when hypertension arises, it can be classified into either early-onset (EO, <34 weeks gestation) or late-onset (LO, ≥34 weeks gestation) subtypes [5]. Hypertension, as the main criteria in a diagnosis of PE, is strongly associated with the initiation and progression of PE. There are two types of hypertension. Gestational hypertension occurs after 20 weeks of pregnancy in the absence of other symptoms of PE. Chronic hypertension occurs before pregnancy of other causes rather than pregnancy itself and is associated with the development of superimposed PE [6].

Cord blood refers to the blood remains in the placenta and the umbilical cord after delivery. Thus, it has been considered a valuable source to study if PE has adverse effects on the placenta and if these can be delivered to the fetus. As previously reported, PE is associated with abnormalities in the cord blood in different aspects, including changes in Biochemistry, immune cell subtypes, Hematology, and Epigenetics [7,8,9,10]. In addition, fetuses exposed to PE havean increased risk of neonatal death, cardiovascular diseases [11], neurodevelopmental disorders [12], thrombocytopenia [13], and hypertension [14]. However, the underlying molecular mechanisms of PE and the corresponding regulatory programs are still largely unclear.

It has been reported that PE is associated with enhanced oxidative stress (OS) [15] and inflammatory response [16] contributed by distinct T helper cell subtypes [7]. Th2 and Treg cells arelargely diminished in PE compared to normal pregnancies. The shift in Th1/Th2 and Th17/Treg balance may further promote inflammation in the umbilical cord blood in PE [8]. RNA-sequencing/microarray was performed using placenta and plasma cell-free RNA to study pregnancies complicated by PE at the transcriptomic level [15,17]. Up-regulated genes in PE are associated with cell proliferation and differentiation, immunity and defense, cell structure, and lipid metabolism and transport, compared to healthy donors. Several genes were suggested as early markers for PE detection, including *CLDN7*, *PAPPA2*, *SNORD14A*, *PLEKHH1*, *MAGEA10*, *TLE6*, and *FABP1*, identified from plasma samples. Higher up in the hierarchy, gene expression during the development and progression of PE is regulated by epigenetic mechanisms, including DNA methylation, histone modification, and microRNAs (miRNAs) [18]. However, investigations on the epigenetic changes in PE are still limited. DNA methylation landscape isthe best-studied epigenetic mechanism in PE [19,20]. Genome-scale DNA methylation profile of cord blood samples from patients with early on-set PE revealed a trend of hypomethylation. Differential expression analysis identified a group of genes involved in inflammation, lipid metabolism, and proliferation, including *RUNX3*, *LINE-1*, *ADORA2B*, *VHL*, *MPP-9*, *TBXAS1*, *ERVW-1*, *SPESP1*, *WNT2*, *AGT*, *DDAH1*, *CALCA*, *IL12B*, *FAS*, *PIK31*, *IGF1*, *GNAS*, *IGF2*, and *HSD11B2* [18,19,21].

Assay for Transposase-Accessible Chromatin with high-throughput sequencing (ATAC-seq) is a method for mapping chromatin accessibility genome-wide [22]. Compared to DNA methylation profiling, ATAC-Seq gives us a more precise overview of open chromatin regions and the underlying gene regulatory networks by peak calling since chromatin accessibility is determined by a dynamic interplay among histones, TFs, and active chromatin remodelers [23]. Thus, we performed ATAC-Seq on cord blood samples from three healthy pregnant donors and a patient with superimposed PE with high sequencing coverage (~33 k reads/sample on average). We identified genes associated with immunomodulation and hypoxia response that have higher chromatin accessibility close to their transcription start sites. Motif analysis indicated that the *GATA* family transcription factor binding was enriched in PE and may play an important regulatory role. Overall, our findings provide an overview of gene regulatory programs and the corresponding downstream pathways associated with PE that may influence the placenta function and fetal growth. This study will be used as a preliminary investigation of a large-scale epigenetic profiling study of PE with different subtypes.

## 2. Materials and Methods

### 2.1. Patient Recruitment Criteria

This study has been approved by the Ethics Committee of the University of Chinese Academy of Sciences Shenzhen Hospital. The research adhered to the tenets of the Declaration of Helsinki as amended in 2013 [24]. All participants recruited have gone through the informed consent process and agreed to donate their cord blood samples. To minimize interpersonal variability, participants were carefully chosen as follows:

PE group—excluded if met any of the following criteria: abnormal pregnancy; gestational diabetes mellitus (GDM); severe organ dysfunction, such as liver, kidney, and heart; co-infection or other inflammatory diseases; history of abnormal pregnancy or delivery (including spontaneous abortion, stillbirth, malformation, etc.); a family history of psychosis or mental illness; a history of alcohol or drug abuse.

Control group: healthy pregnant women who participated in the prenatal examination on time and met the above exclusion criteria with age ≤ 35.

### 2.2. Sample Preparation for ATAC-seq

Cord blood (approximately 50–75 mL) was collected from each donor during delivery. Lymphoprep (Serumwerk Bernburg cat. 1858) was used to isolate mononuclear cells (MNCs) from the cord blood. Red blood cells were lysed using RBC lysing buffer (Biolegend cat. 420310). Cells were recovered from liquid nitrogen. Approximately 10 K cells were processed following the established ATAC-Seq protocol [25]. Samples were sequenced using the MGI2000 platform.

### 2.3. Peak Calling

Raw ATAC-seq reads were mapped to the hg38 reference genome (BioProject: PRJNA31257) using Bowtie (version: 1.3.1). Duplicates were removed with the MarkDuplicates function from Picard Tools (version: 2.26.11) with default parameters. Low-quality reads with mapping quality (mapQ) lower than 30 were removed using samtools. Reads mapped to ChrX, Y, and Random were filtered. MACS2 (version: 2.2.7.1) was used for peak calling with parameters -nomodel -shift -100 -extsize 200. The blacklisted genomic (obtained from https://github.com/Boyle-Lab/Blacklist/blob/master/lists/hg38-blacklist.v2.bed.gz, accessed on 3 March 2022) regions were removed from the peak files using bedtools. Peaks from all samples were merged using bedtools. HTseq-count (version: 1.99.2) was used for counting reads within each peak. Then R package ‘EdgeR’ was used to identify differentially expressed peaks with a false discovery rate of less than 0.1 and |log fold change (logFC)| > 2. To identify peaks with high confidence, we further strengthened the selection criteria by adding an additional cutoff of log count per million (logCPM) > 0.

### 2.4. Peak Annotation

The annotatePeaks.pl function of HOMER [26] (version: 4.11.1) was utilized to associate peaks with nearby genes and to annotate the genomic location of each peak.

### 2.5. Peak Visualization

Integrative Genomics Viewer (IGV) [27] (2.12.3) was utilized to visualize the peaks.

### 2.6. Pathway Enrichment Analysis

The Kyoto Encyclopedia of Genes and Genomes (KEGG) pathway enrichment analysis was performed by KOBAS [28] (http://kobas.cbi.pku.edu.cn/, accessed on 28 March 2022). Enriched pathways were selected as FDR < 0.1(calculated by hypergeometric test/Fisher’s exact test).

## 3. Results

### 3.1. Case Presentation

The patient with PE recruited in this study was a 30-year-old pregnant woman with a history of chronic hypertension for 8 years who developed chronic hypertension associated with superimposed PE with proteinuria at 35 weeks of pregnancy. Before labor, the last blood test results showed abnormal coagulation (PT: 9.3 s, FIB: 3.88 g/L, PT-R: 121.1% and D-Dimer: 0.79 mg /L). However, proteinuria and most of the abnormal blood test results were improved after delivery. (Table 1). To understand if PE would induce any epigenetic changes that may impact the placenta function and fetal growth, we performed ATAC-seq, which allowed us to uncover gene regulatory programs using the cord blood sample from the patient with PE. Three cord blood samples collected from healthy pregnant women were used as negative controls. (Supplementary Table S1; The main test result of healthy gravidae (before labor)).

### 3.2. Immune, Myocardiopathy, and Hypoxia Response-Related Pathways Are Up-Regulated in Superimposed PE

ATAC-Seq identifies the gnome-wide set of cis-regulatory elements and transcription factor binding profiles from open chromatin regions using a hyperactive Tn5 transposase that cuts and ligates sequencing adaptor into the regions. Hence, these regions are enriched with sequencing fragments that are also referred to as peaks. MACS2 is a widely used peak calling tool that defines regions where transcription factors bind.

In this study, 34,304 peaks were identified from the four ATAC-Seq samples using MACS2. Although peak intensities of the four samples at TSS were similar in general (Supplementary Figure S1A,B), differential expression analysis, using edgeR with cutoffs of |logFC| > 2 and FDR < 0.1, identified 786 differential expressed peaks comparing between the patient with superimposed PE and the three healthy controls, among which 767 were up-regulated and 19 were down-regulated. (Supplementary Figure S1C). Peaks were then mapped to their closest genes based on the peak-to-TSS (Transcription Start Site, TSS) distances. 

**Figure 1:**
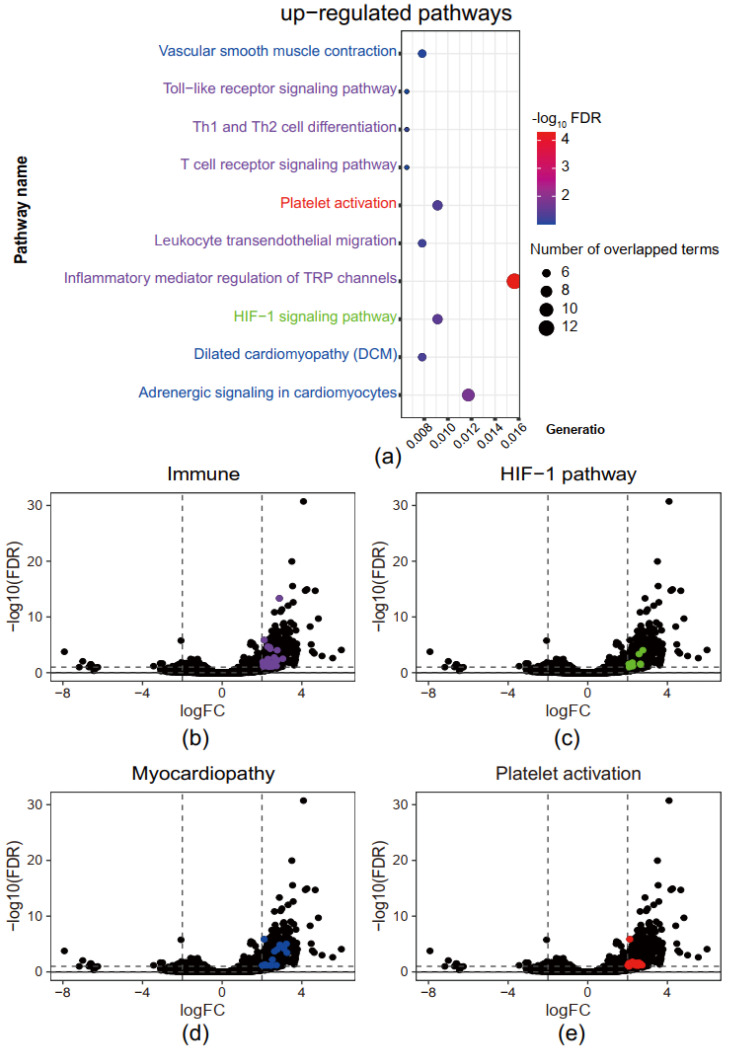
Immune, myocardiopathy, and hypoxia response-related pathways are up-regulated in superimposed PE (**a**) Kyoto Encyclopedia of Genes and Genomes (KEGG) pathway enrichment analysis of genes with higher chromatin accessibility (**b**–**e**) The volcano plot of immune, hypoxia response, hypertension, and platelet activation related gene. HIF-1 stands for Hypoxia Inducible Factor-1.

To further investigate the biological functions of genes with higher chromatin accessibility at their TSS in PE, we conducted pathway analysis using the KEGG database to annotate the up-regulated genes. Only unique genes were extracted for the pathway analysis. Results indicated that the enriched pathways fell into four broad groups: (1) immune (in purple), including Th1 and Th2 cell differentiation, T cell receptor signaling, Toll-like receptor signaling, leukocyte transendothelial migration, and inflammatory mediator regulation of TRP channels; (2) myocardiopathy (in blue), including vascular smooth muscle contraction, dilated cardiomyopathy and adrenergic signaling in cardiomyocytes); (3) hematopoiesis (in red), including Platelet activation; and (4) hypoxia response (in green), including HIF-1 signaling (Figure 1a). Full lists of pathways with adjusted *p*-values are available in Supplementary Table S2 (up-regulated pathway). Genes contributing to these four pathways were highlighted in the same color, respectively (Figure 1b–e). 

### 3.3. Higher Chromatin Accessibility across TSS of Genes of Differentially Expressed Peaks in Superimposed PE

**Figure 2:**
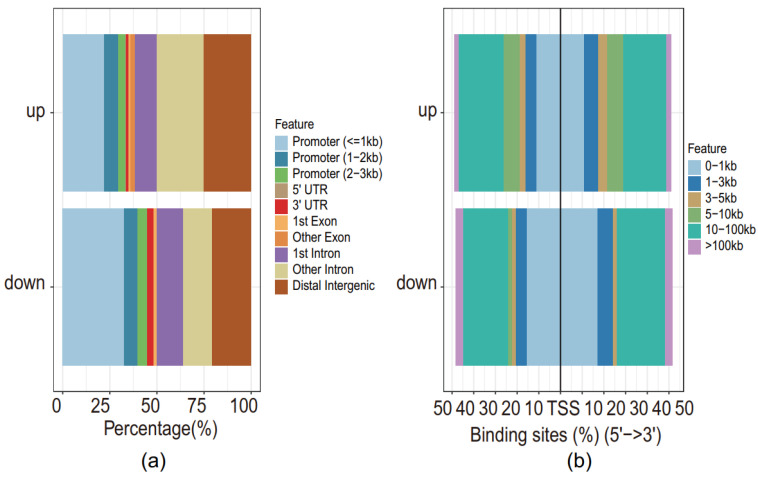
Genomic locations of the differentially expressed peaks. (**a**) Proportions of genomic locations. UTR stands for the untranslated region. (**b**) Proportions of peak-to-TSS distances.

We further investigated the genomic locations of the 786 differentially expressed peaks as detected above. Since these peaks represent either cis-regulatory elements or transcription factor binding sites, they may locate in different regions relative to a gene:(1) Promoter (≤1 kb): less than 1 kilobase (kb) away from the TSS; (2) Promoter (1–2 kb): 1–2 kb away from the TSS; (3) Promoter (2–3 kb): 2–3 kb away from the TSS; (4) 5′ UTR: untranslated region (UTR) at the 5′ of a gene; (5) 3′ UTR: UTR at the 3′ of a gene; (6) 1st Exon: the first exon of a gene; (7) Other Exon: other exons apart from the first one; (8) 1st Intron: the first intron of a gene; (9) Other Intron: other introns apart from the first one; (10) Distal Intergenic: the distal intergenic regions. Thus, each peak was annotated with a unique genomic location. More than 50% of the peaks, either up-regulated or down-regulated, were at the non-promoter regions, indicating potential cis-regulatory element binding sites, which could either be enhancers or silencers that regulate genes nearby (Figure 2a,b).

### 3.4. GATA-Binding Motifs Are Enriched in Superimposed PE

**Figure 3:**
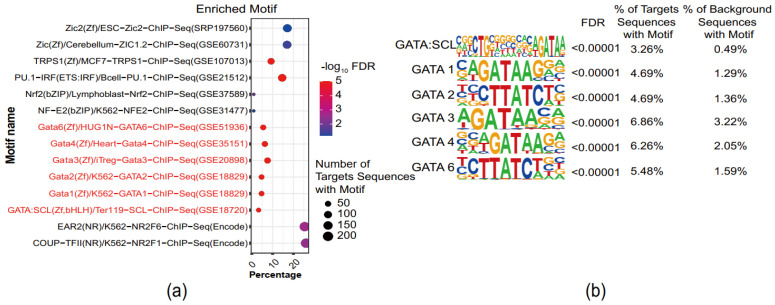
GATA-binding motifs are enriched in superimposed PE (**a**) The enriched motifs. The analysis was conducted by HOMER with parameters set to ‘find regulatory element’. (**b**) The enriched *GATA* family motifs.

HOMER contains a motif discovery algorithm that was designed for regulatory element analysis in genomics applications [26], which can be applied to nucleic acid sequences. Thus, it was used to identify transcription factors that may have a regulatory role in the progression of PE by finding the enriched motifs in the defined open chromatin regions that were only detected in the PE sample. The results revealed enrichment of five *GATA* family transcription factors—*GATA* 1,2,3,4,6 and a co-binding motif of both *GATA* and *SCL*. (Figure 3). We have extracted the peak regions in various types of blood cells from the available ChIP-Seq/ChIP-ChIP datasets of *GATA* factors, predominately *GATA1* and *GATA2*. Associating these peaks within nearby genes, we confirmed that the *GATA* family binds to different genomic locations, among which the majority are not located inthe promoter regions [29,30,31,32,33,34] (Supplementary Figure S2), indicating that the *GATA*transcription factors were able to bind to canonical *GATA* sequence motifs at *cis*-regulatory regions of many PE-associated genes during the disease progression.

### 3.5. Identification of Novel PE-Associated Genes

**Figure 4:**
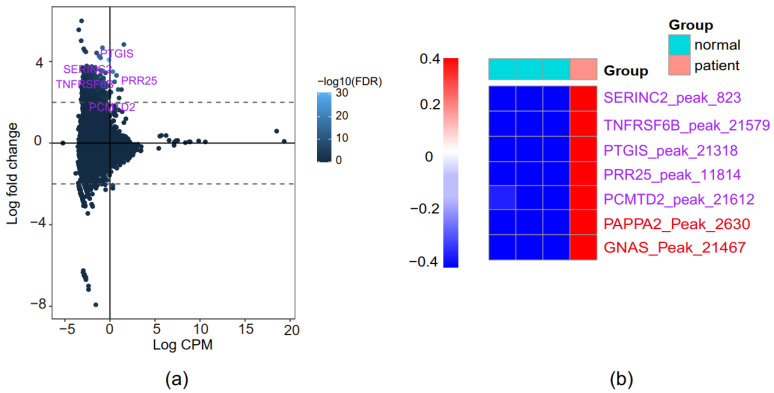
Identification of novel PE-associated genes (**a**) The M-versus-A plot (MA plot) of peaks. (**b**) Heatmap of possible PE-associated genes.

We further filtered the differentially expressed peaks with an additional cutoff of logCPM> 0. As expected, only a few peaks remained but were considered with higher confidence in terms of reproducibility. There were eight peaks up-regulated, among which five are close to TSS of five protein-coding genes—*PTGIS*, *SERINC2*, *PRR25*, *TNFRSF6B*, and *PCMTD2*, that have not been previously reported in PE (Figure 4a). *PTGIS* is a well-known marker gene for cardiovascular diseases and hypertension. *PRR25* is involved in cardiomyopathy. *SERINC2*, *TNFRSF6B*, and *PCMTD2* are associated with immune and pro-inflammatory responses (Supplementary Figure S3). In addition, we observed a similar expression pattern of well-known PE-associated genes, including *PAPPA2* and *GNAS*, although they were not selected as high confidence (Figure 4b). 

## 4. Discussion

PE is a common hypertensive disorder in 2–8% of pregnancies and may lead to severe outcomes, even maternal and fetal deaths. Fetuses after exposure to PE are at a higher risk of developing cardiovascular diseases [11] and various neurodevelopmental disorders, particularly autism spectrum disorder and attention-deficit/hyperactivity disorder (ADHD) [12], attributed to oxidative stress and inflammation. Thus, an in-depth understanding of the underlying molecular mechanisms, especially the transcriptional regulation, is urgently needed to prevent PE and provide further guidance in treating PE and PE-associated disorders. Cord blood collected from the umbilical cord after delivery provides an environment shared between the placenta and the fetus. Thus, it has been considered a valuable source to study if PE has adverse effects on the placenta and if these can be delivered to the fetus. Hence, ATAC-seq was administrated to reveal the epigenetic changes in cord blood from a patient with PE and to interrogate the underlying gene regulatory programs.

Previous studies have already claimed the association of hypoxia with PE. *HIF-1α*, as an oxygen sensor that regulates oxygen homeostasis in the human placenta, plays a key role in the pathogenesis of PE [35,36,37]. Furthermore, maternal PE can also result in fetal hypoxia [38]. An accumulation of pro-inflammatory cytokines, an increase in reactive oxygen species (ROS), and a reduced concentration of antioxidants and antioxidant enzymes in PE have been reported, indicating enhanced inflammation and oxidative stress [12]. Our ATAC-seq data showed higher chromatin accessibility of hypoxia response-related genes in the patient with PE. This points to hypoxia in PE in the cord blood, which may further pass to the fetus under the regulation of the HIF-1 pathway. In terms of cardiovascular function, the up-regulation of Dilated cardiomyopathy (DCM), vascular smooth muscle contraction, and Adrenergic signaling pathways may be owing to uncontrolled hypertension that leads to damaged and narrowed coronary arteries, heart attack, or even heart failure.

Intriguingly, our results showed an up-regulation of the platelet activation pathway in the cord blood from the epigenetic landscape, which is consistent with abnormal coagulation, as indicated in the patient’s regular blood test result. It has been reported that platelet activation is involved in the progression of PE and contributes to the thrombotic and coagulopathic complications of the disease [39,40]. Our results confirmed this statement and provided further evidence that the platelet activation itself can potentially be used as a biomarker for the prediction of PE.

Altered immunomodulation is the first pathogenic sign of PE, which refers to an abnormal immune response to the allogeneic fetus [41,42,43]. Pathway enrichment analysis discovered an enrichment of immune response pathways that involved various subtypes of T helper cells. T helper cells are CD4 positive lymphocytes that can differentiate into several subtypes, including Th1, Th2, Th17, etc. These subtypes modulate immune responses by secreting a different panel of cytokines that trigger or suppress inflammation. It has been found that the Th1/Th2 balance is altered in PE, which is characterized by a higher ratio of circulating Th1/Th2 lymphocytes [44]. Our ATAC-Seq results also provided a hint of changes in the T helper cell subtype ratios. We found that the T helper cell subtype differentiation-related pathway and the Th1 and Th2 cell differentiation pathways were up-regulated in PE, which is in line with previous findings [41,42,43]. In addition, immune response pathways, including Toll-like receptor signaling pathway, T cell receptor signaling pathway, and Inflammatory mediator regulation of TRP channels, were observed, suggesting a pro-inflammatory outcome contributed by the imbalanced subtypes of T helper cells. We also detected an up-regulation of leukocyte transendothelial migration in PE, indicating an inflammatory infiltration. All the above results reflect an inflammatory microenvironment of cord blood in PE.

Furthermore, motif analysis of the up-regulated peak regions revealed enrichment of *GATA* family transcription factors. *GATA* factors are associated with the development and differentiation of T helper cells and other types of immune cells, such as dendritic cells and macrophages [45]. The *GATA* family, especially *GATA3*, regulates Th1/Th2 cell differentiation [46,47]. In addition, the GATA family is also associated with the hypoxia response via HIF-1 pathways. *GATA3* has the ability to interact with and stabilize HIF-1α protein under the hypoxia condition, resulting in prolonged hypoxia response [48]. Thus, the *GATA* family, including *GATA1*, *GATA2*, *GATA3*, and *GATA4*, are likely to be key regulatory elements that regulate the differentially expressed genes involved in the pathogenesis of PE.

**Figure 5:**
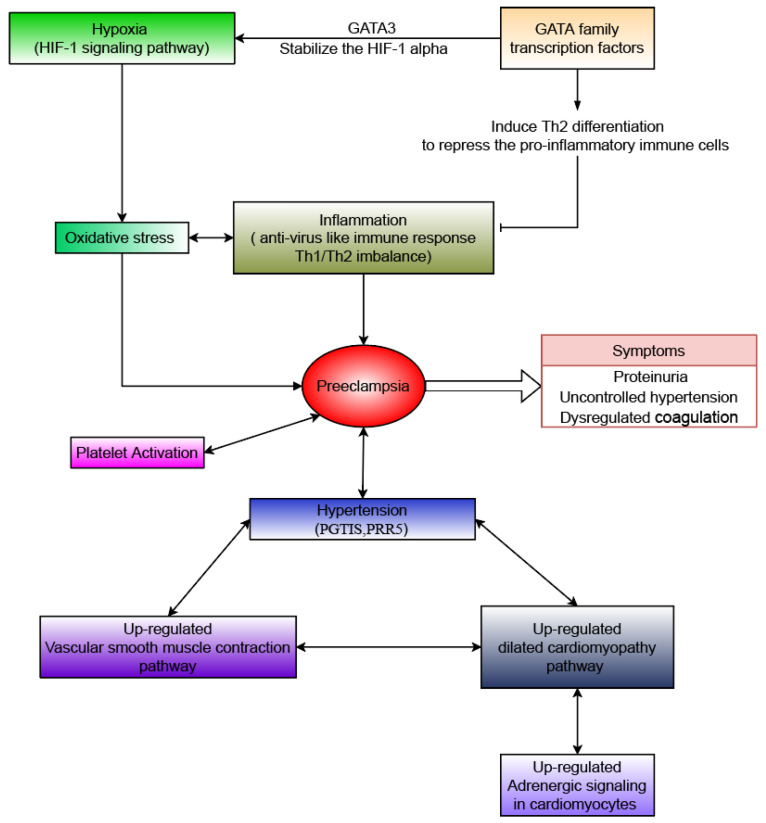
The mechanism map.

In agreement with previous studies, our findings provide further evidence that hypoxia, oxidative stress, inflammation, and cardiovascular dysfunction are mutually linked, resulting in the pathophysiological progression of PE (Figure 5). Although the number of samples involved in this study is limited, especially for the PE condition, our ATAC-seq results provided an overview of gene regulatory programs and the corresponding downstream pathways associated with PE that may influence placenta function and fetal growth. Additionally, we discovered novel genes with high confidence that were not previously reported and could be potentially used as biomarkers for the prediction and phenotyping of PE. This case study can be used as a preliminary input and provides guidance to any future large-scale epigenetic profiling studies of PE.

## 5. Conclusions

Our ATAC-seq results provide an overview of gene regulatory programs and the corresponding downstream pathways associated with PE that may influence placenta function and fetal growth. In addition, we discovered novel genes with high confidence that were not previously reported and could be potentially used as biomarkers for the prediction and phenotyping of PE.

## Figures and Tables

**Table 1:** The main abnormal test result of the patient.

	Before Labor	After Labor	Reference Range
**Urine**			
Protein	+ +	−	−
24 h urine protein (mg/24 h)	876.58 ↑↑	−	<150
**Blood** *			
BNP (pg/mL)	909 ↑↑	NA	<100
PT (s)	9.3 ↓	10.3	10~15
FIB (g/L)	3.88 ↑	5.75 ↑	1.8~3.5
PT-R (%)	121.10 ↑	111.70	75~120
D-D (mg/L)	0.79 ↑	1.12 ↑	<0.5

* BNP stands for B-type Natriuretic Peptide. PT stands for prothrombin time. FIB stands for fibrinogen. PT-R stands for prothrombin time activity. D-D stands for D-Dimer. ↑ means higher than normal value. ↓ means lower than normal value. + means positive, − means negative.

## Data Availability

The ATAC-seq sequence is available in GSE199479. ChIP-Seq data of GATA family transcriptional factors binding sites were obtained from GSM970258, GSM1278240, GSM970257, GSM651546, GSM651547, GSM1097883, GSM1816080, GSM607949, GSM607950.

## Supplementary Materials

The following supporting information can be downloaded at: https://www.mdpi.com/article/10.3390/genes13050888/s1. The main test results of healthy gravidae (before labor) are submitted as Table S1. The lists of pathways and differentially expressed peaks are submitted as Table S2 and Table S3, respectively. The heatmap of distribution of transcription factor binding loci relative to TSS, the distribution of transcription factor binding loci relative to TSS, and volcano plot of differential expressed genes are submitted as Figure S1A–C, respectively. The binding site distribution is presented in Figure S2; the ChIP-Seq data were obtained from GSM970258, GSM1278240, GSM970257, GSM651546, GSM651547, GSM1097883, GSM1816080, GSM607949, GSM607950. All bed files were merged into one bed file and then were annotated. Peaks across the promoter regions of *PGTIS*, *SERINC2*, *PCMTD2*, *TNFRSF6B*, and *PRR25* are presented in Figure S3.
