# Supplementary material for: Preliminary Interpretations of Epigenetic Profiling of Cord Blood in Preeclampsia
Source: Genes (Basel). 2022 May 16;13(5):888. doi: 10.3390/genes13050888 (PMC9141867; doi:10.3390/genes13050888)

Supplementary Figure

Supplementary Figure S1

(A) The heatmap of distribution of transcription factor binding loci relative to TSS
(B) The Distribution of transcription factor binding loci relative to TSS
(C) Volcano plot of differential expressed genes

Supplementary Figure S2

(A) The Distribution of GATA family transcription factor binding sites

Supplementary Figure S3

(A-E) Peaks across the promoter regions of PGTIS , SERINC2, PCMTD2, TNFRSF6B and PRR25

Supplmentary Figrue S1

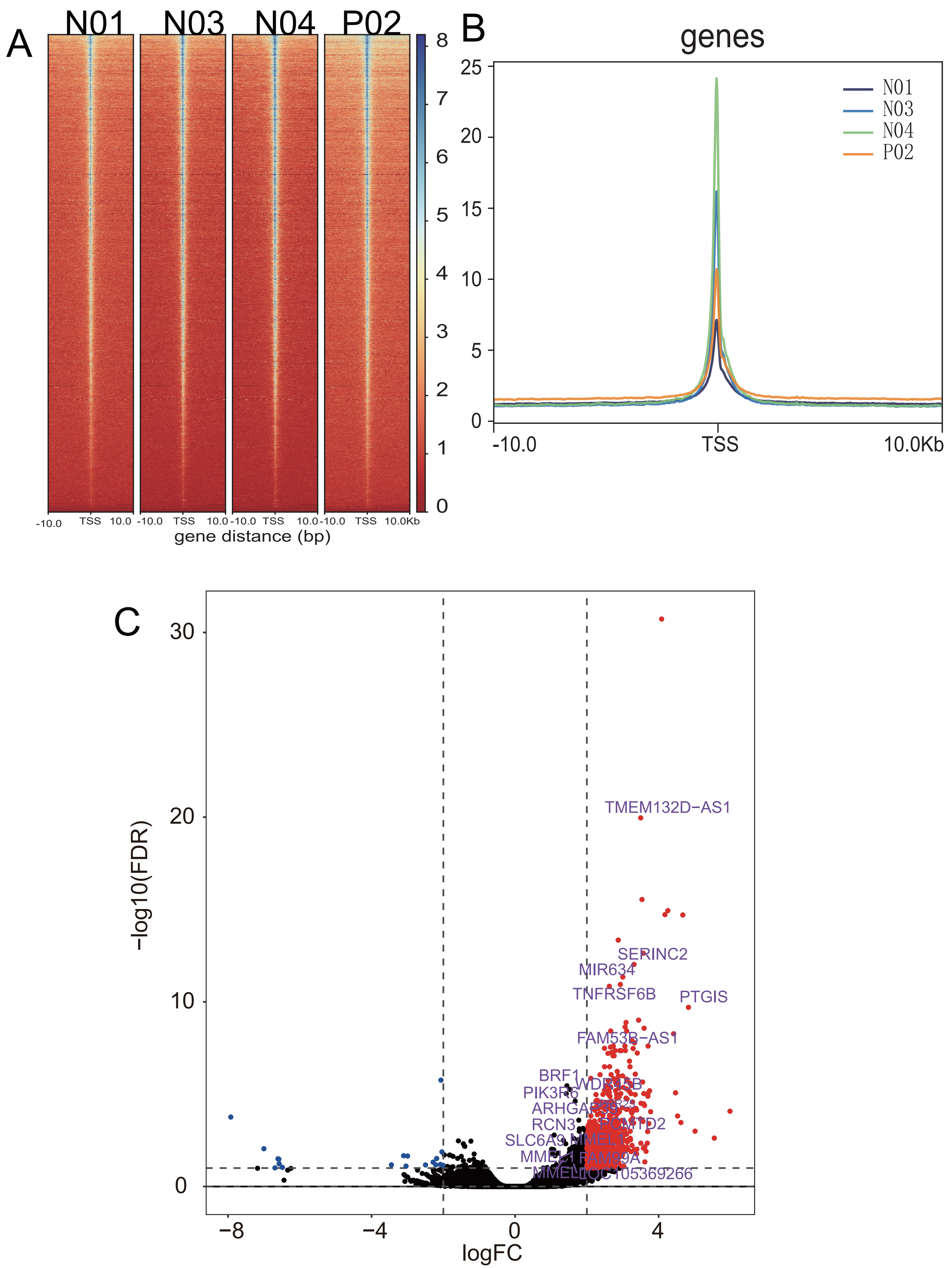

Supplmentary Figrue S2

A

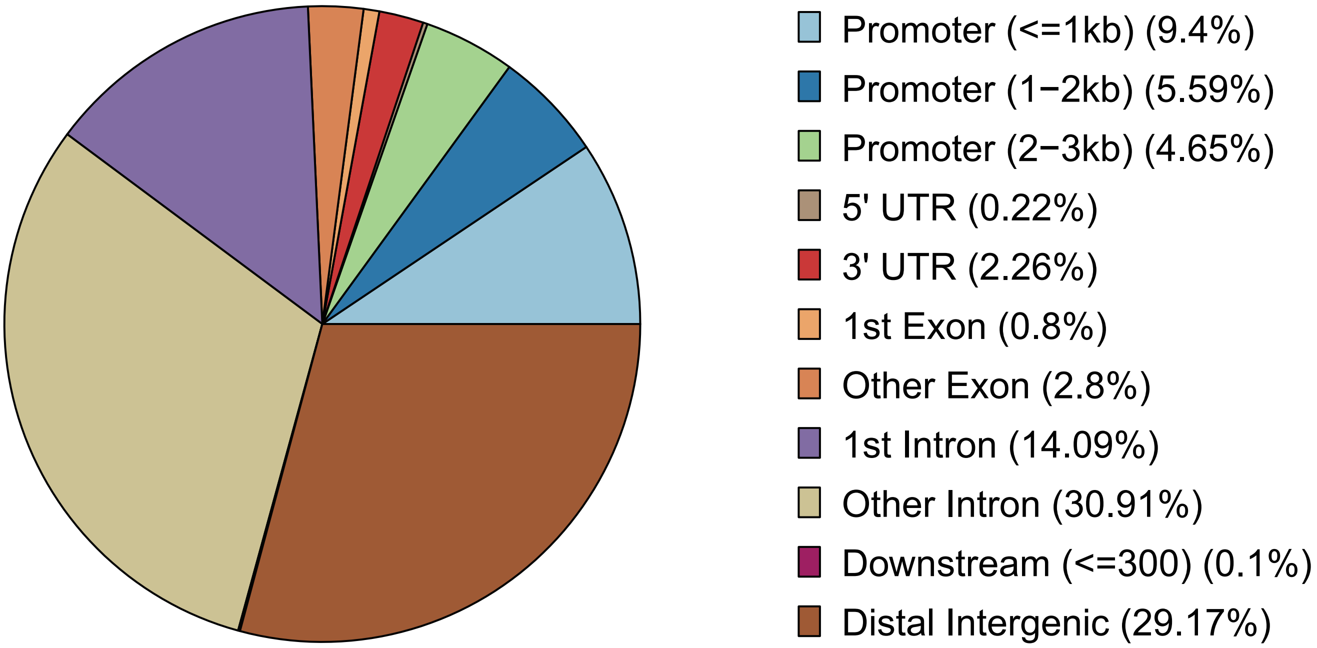

Supplmentary Figrue S3

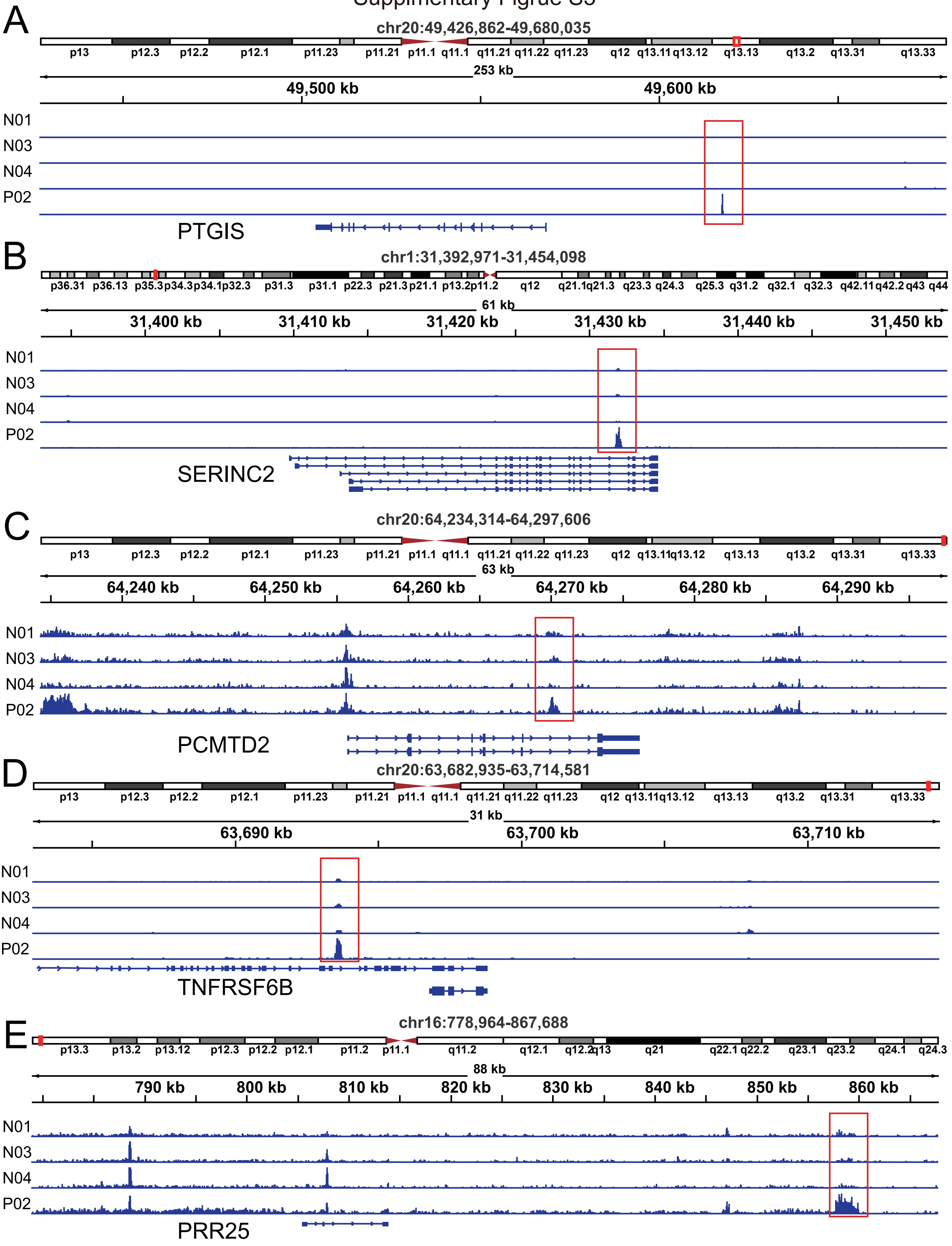

Supplement: Supplementary file 1 [file genes-13-00888-s001.zip › Supplementary Figures.pdf]
