# Supplementary material for: Preliminary Interpretations of Epigenetic Profiling of Cord Blood in Preeclampsia
Source: Genes (Basel). 2022 May 16;13(5):888. doi: 10.3390/genes13050888 (PMC9141867; doi:10.3390/genes13050888)
Supplement: Supplementary file 1 [file genes-13-00888-s001.zip › Supplementary Table S1 The main test result of healthy gravidae(before labor).pdf]

Supplementary Table S1

Supplementary Table S1	The main test result of healthy gravidae (before labor)		
	N01	N03	N04
Urine			
Protein	-	-	-
24h urine protein	-	-	-
Blood			
BNP	NA	NA	NA
PT	11.2 s	11 s	11.1 s
FIB	6.91 g/L ↑	5.44 g/L ↑	3.31 g/L
PT-R	97.4 %	100.3 %	98.9 %
D-Dimer	1.15 mg/L ↑	3.82 mg/L ↑	5.91 mg/L ↑

*BNP stands for B-type Natriuretic Peptide. PT stands for prothrombin time. FIB stands for fibrinogen. PT-R stands for prothrombin time activity. D-D stands for D-Dimer. ↑ means higher than normal value. + means positive, - means negative.
